# The Effects of Different Warm-Ups on Volleyball Performance

**DOI:** 10.3390/sports14060218

**Published:** 2026-05-26

**Authors:** Milosz Mielniczek, Roland van den Tillaar

**Affiliations:** Department of Sports Sciences and Physical Education, Nord University, 7600 Levanger, Norway; milosz.msg@gmail.com

**Keywords:** volleyball, warm-up, physical performance, jump performance

## Abstract

Objective: The objective was to evaluate the effectiveness of various warm-up strategies on volleyball-specific physical performance. Background: Warm-ups in volleyball aim to enhance performance and reduce injury risk, but no standardized approach exists and evidence on effectiveness is inconsistent. Methods: A systematic search of PubMed and Google Scholar was conducted through 12 November 2025. Two reviewers independently screened records and assessed risk of bias. A systematic search identified 108 records; 13 met inclusion criteria. Eligible studies examined the effects of different warm-up methods on volleyball-related physical performance. Risk of bias was assessed using the Cochrane Risk of Bias 2 (RoB 2) tool and visualized with the robvis package. Warm-up protocols were categorized into dynamic and static stretching, resisted warm-ups, foam rolling and vibration techniques, whole-body vibration, Raise Activate Mobilize Potentiate (RAMP), joint distraction methods, and volleyball-specific routines. Results: Dynamic, resisted, high-intensity, volleyball-specific, and whole-body vibration warm-ups showed the most consistent improvements in jump performance, agility, and reaction time. Static stretching provided minimal benefits, with occasional gains in agility or flexibility. Foam rolling and vibration foam rolling were largely ineffective, except for one study showing improved reactive strength. The results varied due to differences in athlete level, protocol duration/intensity, sample size, and measurement methods. Conclusion: Warm-ups appear to meaningfully influence volleyball performance. Dynamic, resisted, and sport-specific routines appear to be the most effective. More research is needed to define optimal, standardized protocols.

## 1. Introduction

Warm-up is a crucial preparatory component before training or competition. One of its central effects is the elevation of muscle temperature, which accelerates metabolic reactions, improves oxygen delivery, and enhances the efficiency of both aerobic and anaerobic energy pathways [1,2]. Increasing muscle temperature also enhances the speed of neural conduction and decreases musculotendinous stiffness, which can support force transmission during explosive movements [1,2]. However, this relationship is non-linear and prolonged static stretching (typically >60 s per muscle group) can render the muscle–tendon unit excessively compliant, dissipating energy during the stretch-shortening cycle and impairing rapid force production [3].

Evidence from team sports indicates that when warm-up is skipped, the musculotendinous system remains stiffer and less compliant, increasing mechanical stress during explosive actions such as jumping, rapid acceleration, and changes in direction [4]. Dynamic warm-up research further shows that athletes who do not complete an adequate warm-up experience higher rates of strains, sprains, and overuse injuries compared with those engaging in structured warm-up routines [5]. The absence of neuromuscular activation and increased tissue readiness reduces the ability to handle high velocity movements and elevates injury risk [4,5].

Volleyball athletes are exposed to a lot of repetitive loads, especially on the shoulder, due to the high volume of overhead actions such as spiking and serving. These demands contribute to increased injury risk [6]. When such explosive movements are performed without adequate warm-up, mechanical stress rises and neuromuscular readiness decreases. Because these actions directly influence performance and injury risk, an effective warm-up is particularly important in volleyball.

The literature indirectly but consistently suggests that lower-level players often perform general warm-ups with reduced technical precision and engagement. Gouttebarge, et al. [7] found that a structured warm-up intervention was needed, as existing ones lacked injury prevention components like stability, strength, and balance exercises in adult recreational volleyball teams. The resulting “VolleyVeilig” warm-up program, designed to fill this gap, reduced acute injuries by about 21% in a 672-player randomized controlled trial (RCT) and cut acute and upper-extremity injuries by 30–59% in youth players, suggesting the control groups’ usual warm-ups were substantially less protective [8,9]. Together, these differences illustrate how competitive-level warm-up shapes the demands placed on athletes during warm-ups and underscore the importance of identifying level-appropriate strategies that effectively support volleyball performance and injury prevention.

Structured warm-up routines have been shown to improve performance and reduce injury risk across several sports. In football, the FIFA 11+ program is one of the most widely validated examples, demonstrating substantial reductions in injury rates when performed consistently [10]. Its success illustrates that well-designed, sport-specific warm-ups can meaningfully enhance readiness and resilience. While such programs have been developed in soccer, volleyball currently lacks a comparable standardized warm-up protocol targeting physical performance, which forms the rationale for the present review.

In volleyball, early evidence also suggests that targeted preparatory approaches can influence performance. An eight-week-in-season wearable resistance intervention improved countermovement jump (CMJ) height in female players, indicating that specific activation strategies can enhance explosive movements [11]. However, a similar eight-week intervention applying wearable resistance to the forearms did not improve performance, underscoring that warm-up effects are highly task specific and must match the biomechanical demands of the skill being targeted [12]. These findings suggest that different warm-up methods can influence volleyball performance, but the optimal structure, duration, and intensity remain unclear.

Despite volleyball’s reliance on explosive performance, rapid directional changes, and high-velocity overhead movements, the sport still lacks a standardized, evidence-based warm-up routine, as reflected by the considerable variation in warm-up protocols observed across the studies included in the present review. To assess warm-up effectiveness, volleyball performance must be defined by measurable outcomes such as jump height, change in direction speed, and ball velocity in serving or spiking, qualities that directly affect attacking options, blocking reach and opponent pressure.

This gap in the literature led us to investigate whether there is clear evidence identifying which warm-up strategies can effectively enhance volleyball performance and to evaluate how the different approaches used in existing studies influence key performance outcomes.

## 2. Materials and Methods

### 2.1. Eligibility Criteria

A set of inclusion and exclusion criteria, presented in Table 1 below, was applied to ensure that the findings of this review were relevant, comparable, and methodologically sound. Studies were eligible for inclusion if they met the following criteria: (1) involved volleyball players, (2) used warm-up as the primary intervention, and (3) assessed at least one volleyball-relevant physical or technical performance outcome. Acceptable performance measures included vertical jump height, CMJ, approach jump, agility or change in direction tests, sprint performance, spike or serve speed, and spike or serve accuracy. Only peer reviewed studies written in English and with full text availability were considered to ensure transparency and access to methodological details, consistent with previously established criteria. No restrictions were placed on participant sex or age, and studies including mixed-gender cohorts were eligible. Warm-up interventions of any type such as dynamic warm-ups, ballistic or plyometric exercises, post-activation performance enhancement (PAPE) protocols, stretching-based warm-ups, and volleyball-specific warm-ups were included if the protocol was clearly described. Studies were excluded if they met any of the following criteria: (1) focused exclusively on injury prevention without measuring performance outcomes; (2) used warm-up elements as part of a broader training program rather than as a pre-performance intervention; (3) did not include volleyball athletes; or (4) were review papers, meta-analyses, conference abstracts, or case reports. This ensured that only original empirical research directly examining warm-up effects on volleyball performance was synthesized.

### 2.2. Information Sources

A systematic search was conducted using the PubMed and Google Scholar databases. PubMed was selected due to its extensive coverage of peer-reviewed research in sports science, physiology, and related biomedical fields, while Google Scholar was included to capture additional studies from coaching science, biomechanics, and sport-specific journals that may not be indexed in traditional medical databases. These databases together provided a broad yet focused scope for identifying relevant studies. The search was performed on 12 November 2025 with no restrictions on the starting date to ensure that all available literature relevant to the topic was captured. The full-search strategy for all databases included the following terms: (warm-up OR “warm up”) AND volleyball AND (performance OR jump OR agility OR “reaction time”).

### 2.3. Search Strategy

To ensure adequate specificity and avoid retrieving large numbers of irrelevant warm-up studies from other sports, a simple combination of pre-determined keywords was used. The primary search terms included “warm-up” and “volleyball”, which were applied to the title field when possible to increase relevance. This approach allowed for efficient identification of studies directly addressing volleyball-related warm-up interventions. The search terms were applied independently in PubMed and Google Scholar. In PubMed, filters for free full text and English language were used to ensure full accessibility of methodological information. Google Scholar searches were restricted to titles to improve specificity and reduce the inclusion of unrelated literature. No restrictions were placed on publication year to capture all available studies relevant to volleyball performance.

### 2.4. Selection Process

The selection process for this systematic review was conducted in two stages. In the first stage, all records identified through the database searches were screened by title and abstract to assess their relevance to the research question. This initial screening was performed independently by the first author (M.M.), applying the predefined eligibility criteria to remove studies unrelated to volleyball, not focused on warm-up interventions, or lacking performance-based outcome measures. In the second stage, full-text versions of the remaining articles were retrieved and assessed for eligibility. Full-text screening was carried out by M.M. and independently reviewed by R.T. to ensure accuracy and consistency. Discrepancies or uncertain cases were discussed until consensus was reached. The complete selection process, including the number of records identified, screened, excluded, and included in the final synthesis, will be illustrated using a PRISMA flow diagram [13] in the Results section, following standard reporting guidelines. The completed PRISMA checklist is provided in the Supplementary File S1.

### 2.5. Data Collection Process

Data extraction was performed by the first author (M.M.) using a standardized data extraction template developed for this review. Data extraction was performed by the first author (M.M.) and independently verified by the second author (R.v.d.T.) to ensure accuracy and consistency. The template included information on participant characteristics (sample size, age, sex, and competitive playing level), study design, and intervention details (type of warm-up, duration, and intensity). The primary outcomes sought were physical performance measures. These included jump performance, agility/change in direction, sprint performance, and sport-specific measures (e.g., spike or serve performance), along with study and intervention characteristics. All included studies reported warm-up interventions performed immediately before performance testing. Performance outcomes were extracted exactly as reported by the authors, without imputation or modification. Where relevant, additional notes were made regarding testing environment and sport-specific procedures to support accurate interpretation in the narrative synthesis.

### 2.6. Study Risk of Bias Assessment

To ensure methodological rigor, the risk of bias for each included study was assessed independently by two reviewers (M.M. and R.v.d.T.), with any discrepancies resolved through discussion. The risk of bias for each included study was assessed using the Cochrane Risk of Bias 2 (RoB 2) tool and visualized using the RobVis [14] tool, a widely used web-based application designed to visualize risk of bias evaluations within systematic reviews. This tool was selected for its clarity, accessibility, and ability to generate standardized graphical outputs that enhance transparency in reporting. The assessment focused on key methodological domains defined by the RoB 2 tool: bias arising from the randomization process, deviations from intended interventions, missing outcome data, measurement of the outcome, and selection of the reported result. Reporting bias was assessed using Domain 5 of the RoB 2 tool. Each domain was classified as having a “low risk”, “some concerns”, or “high risk” of bias. The results of the risk of bias evaluations are presented using a traffic light plot, illustrating domain level judgments for each study. Certainty of evidence was qualitatively assessed based on study design, consistency of results, and risk of bias judgments.

### 2.7. Synthesis Methods

A narrative synthesis approach was used to analyze and interpret the findings from the included studies. A meta-analysis was not performed due to substantial heterogeneity across studies in (i) outcome measures (e.g., CMJ, drop jump, sprint, agility, ROM, and reaction time, with different units and devices); (ii) warm-up protocols (varying in duration, intensity, exercise selection, and sequencing); (iii) study designs (randomized crossover, repeated measures, and single-group pre- and post-test designs); and (iv) participant characteristics (age, sex, and competitive level ranging from youth to elite). Instead, results were synthesized descriptively, focusing on identifying common patterns, comparing warm-up types, and summarizing their effects on performance.

## 3. Results

### 3.1. Study Selection

**Figure 1:**
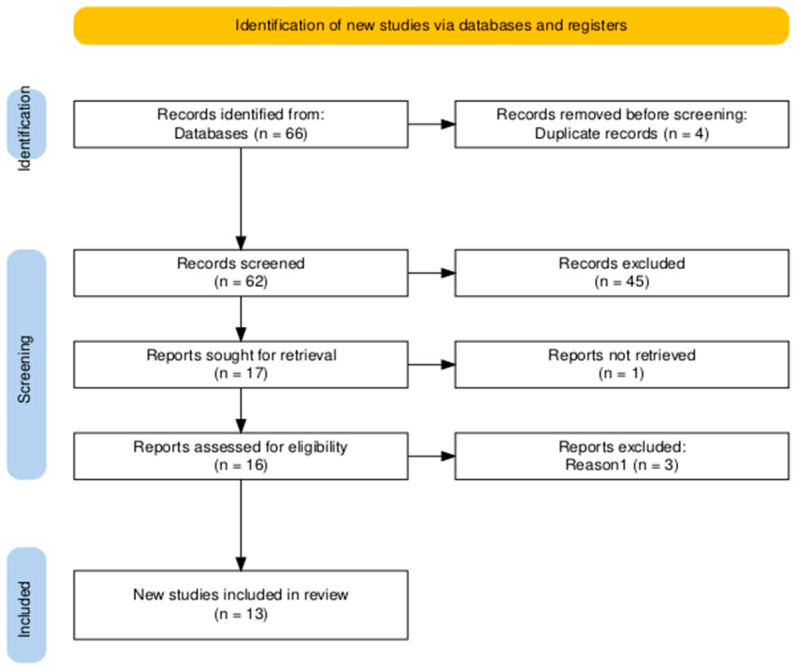
The PRISMA flow diagram of the study selection process.

The database search produced 108 records. After removing duplicates and screening titles and abstracts, 24 articles remained for full-text review. Eleven articles were excluded at the full-text stage for the following reasons: did not include volleyball athletes, did not apply a warm-up intervention, did not measure performance outcomes, or did not meet the criteria for peer-reviewed original empirical research. The PRISMA flow diagram (Figure 1) illustrates the full screening process. A total of 13 studies met all inclusion criteria and were included in this review.

### 3.2. Risk of Bias in Studies

**Figure 2:**
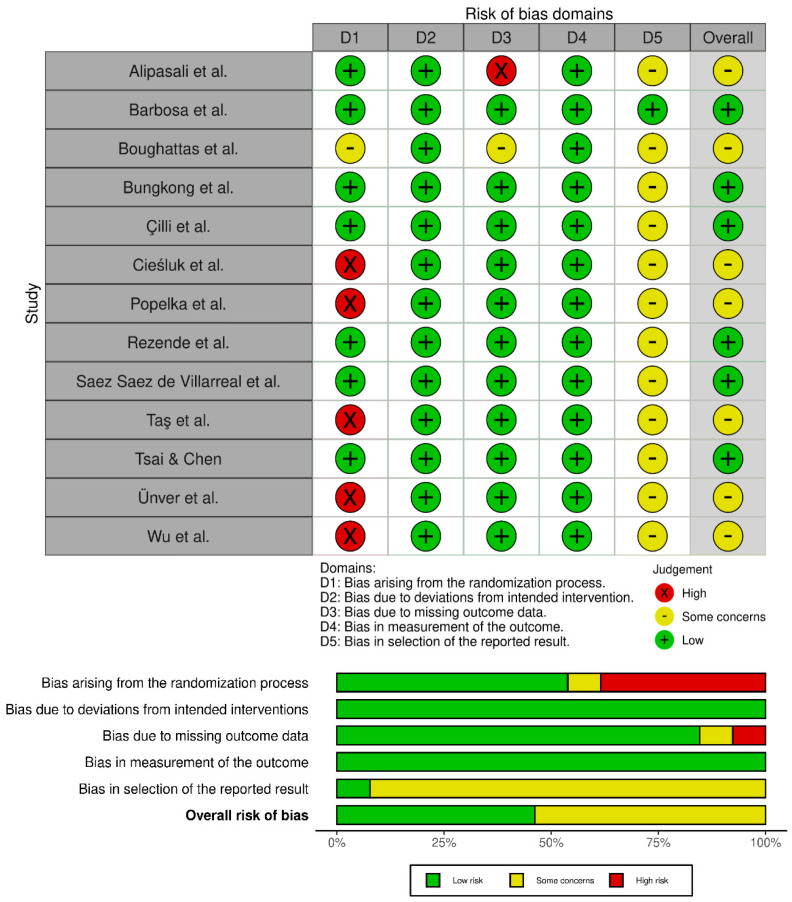
Risk of bias [15,16,17,18,19,20,21,22,23,24,25,26,27].

Risk of bias was evaluated for all 13 included studies using the Cochrane RoB 2 tool, with results visualized through the robvis package (Figure 2), which provides a domain-based visual summary through a “traffic light” plot. The assessment covered five domains: bias arising from the randomization process (D1), deviations from intended interventions (D2), missing outcome data (D3), measurement of outcomes (D4), and selection of reported results (D5). Reporting bias results are integrated into Domain 5. Overall methodological quality varied between studies. Six studies [15,16,17,18,19] were judged to have an overall low risk of bias, primarily due to randomized crossover designs, complete data reporting, and the use of objective measurement tools. Seven studies [20,21,22,23,24,25,26] were rated as having some concerns, typically due to non-randomized designs, absence of preregistration, or insufficient detail regarding reporting decisions. No studies were classified as high risk of bias in the final dataset. Across the included studies, the most frequent methodological issue was the absence of preregistration or publicly available protocols (D5), followed by non-randomized designs (D1). Conversely, deviations from intended interventions (D2), missing outcome data (D3), and outcome measurement procedures (D4) consistently showed low risk across nearly all studies. Overall certainty of evidence was judged as moderate, primarily downgraded due to the general lack of study preregistration.

### 3.3. Study Characteristics

The 13 included studies examined a wide range of warm-up protocols in volleyball athletes, with samples spanning youth, adolescent, collegiate, amateur, and elite adult players. Study designs were mainly randomized crossover or repeated measures, though several studies used single-group pre- and post-test designs. Warm-up interventions varied and included dynamic warm-ups; static stretching; foam rolling and vibration foam rolling; whole body vibration; joint distraction protocols; cycling or resistance-based warm-ups; volleyball-specific routines; Raise, Activate, Mobilize, Potentiate (RAMP) warm-ups; and resisted dynamic warm-ups.

Across studies, performance was assessed using physical and neuromuscular outcomes. Commonly measured variables included vertical jump height, CMJ performance and power output, agility or change in direction, linear sprint speed, flexibility or joint range of motion, reaction time, and drop jump performance. This heterogeneity in warm- up protocols and outcome measures reflects the diverse approaches used to prepare athletes for explosive actions. All characteristics of included studies are presented below in Table 2.

### 3.4. Results of Individual Studies

Across the included studies, dynamic warm-up protocols generally produced superior performance outcomes compared with static stretching. The most consistent improvements were observed in vertical and horizontal jump performance, with one study reporting gains of approximately 3.5% and 4.2%, respectively, following a dynamic warm-up. Static stretching showed limited acute benefits, with one study reporting improved agility, but several others showing no meaningful effect on jump height, power, or speed.

Foam rolling and vibration foam rolling produced mixed results. Most studies reported no significant improvements in jump or reactive strength measures; however, one study found that standard foam rolling produced better drop jump outcomes than vibration foam rolling or passive rest.

Several targeted or combined warm-up approaches produced positive effects. A protocol combining routine warm-up with lower extremity joint distraction improved flexibility, ankle dorsiflexion range of motion, and both vertical and horizontal jump performance. Sport-specific warm-ups, including volleyball-oriented or high-intensity short-duration routines, resulted in boosting vertical jump across sub-elite and elite players. Whole body vibration warm-ups improved agility and speed strength in elite male athletes, while resisted dynamic warm-ups produced significant increases in jump height compared with non-resisted versions.

Warming up consistently outperformed no warm-up across nearly all studies. One study demonstrated that warm-up followed by stretching improved vertical jump more than stretching performed before warm-up, suggesting that the order in which players perform these warm-ups also plays a role on the outcomes. RAMP warm-ups produced meaningful improvements in reaction time in youth players. Long-term warm-up-based stretching interventions (e.g., 6-week dynamic or static stretching programs) improved sprint ability, with no clear superiority between stretching types. Despite these patterns, several studies reported no significant differences among different warm-ups for performance, highlighting variability depending on athlete level, protocol structure, and performance outcome measured.

## 4. Discussion

The main finding of this systematic review is that warm-up strategies that elevate muscle temperature and enhance neuromuscular activation are more likely to improve volleyball-specific performance than strategies that primarily increase range of motion without engaging sufficient neuromuscular drive, that is, without recruiting high-threshold motor units at firing rates capable of supporting explosive force production. This pattern is consistent with established physiological models describing how increased muscle temperature speeds up metabolic reactions, improves neural conduction velocity, and enhances muscle–tendon compliance [1,2], all of which are crucial for explosive actions such as jumping, rapid acceleration, and rapid change in direction movements in volleyball. It should be noted, however, that the relationship between muscle–tendon compliance and explosive performance is not straightforward. Prolonged static stretching has not consistently been shown to enhance explosive performance outcomes [16,21]. The proposed mechanism is that excessive compliance of the muscle–tendon unit may dissipate elastic energy over a longer time window, attenuating the rebound forces produced during the stretch-shortening cycle and reducing rate of force development.

Across the included studies we found that dynamic, resisted, high-intensity, volleyball-specific, and whole-body vibration warm-ups produce the best acute performance benefits. Saez Saez de Villarreal et al. [18] demonstrated that warm-ups incorporating loaded jumps, high-intensity resistance exercises, drop jumps, or a volleyball-specific routine resulted in superior improvements in jump height and power compared with lower-intensity or nonspecific protocols. Similarly, Rezende et al. [16] reported that a specific jump warm-up showed greater CMJ performance in youth players than static stretching, cycling, or the control group. This supports the idea introduced earlier that warm-ups must prepare the stretch-shortening cycle and neural drive to be effective for volleyball performance.

Warm-ups with use of additional resistance or higher intensity appear particularly effective in better trained athletes. Çilli et al. [15] showed that a resisted dynamic warm-up significantly increased jump performance compared with a non-resisted condition, while Ünver et al. [25] reported that a short-duration, higher-intensity warm-up improved jump height in both elite and sub-elite female players. This suggests that better trained athletes benefit from warm-ups that include greater mechanical and neural demands, likely due to their enhanced ability to recruit high-threshold motor units and tolerate higher intensities without fatigue. The mechanisms behind the superior effects of high-intensity and resisted warm-ups can be explained at both the peripheral and central level. At the peripheral level, conditioning contractions trigger phosphorylation of myosin regulatory light chains, which increases the sensitivity of the actin–myosin cross-bridge to calcium and allows for greater force output even at submaximal calcium concentrations [28,29,30]. At the central level, motoneuron excitability increases, reflected in greater recruitment of high-threshold motor units, higher motor unit discharge rates, and changes in persistent inward currents that lower recruitment thresholds [31,32,33]. These combined adaptations are consistent with what is described as post-activation performance enhancement (PAPE) and may help explain why trained athletes, who are better able to recruit and sustain high-threshold motor units, tend to benefit more from higher-intensity warm-up protocols [32,34].

Whole-body vibration gave only some performance benefits in elite volleyball players. Wu et al. [26] reported an increase in block related agility following a brief vibration warm-up, with performance improving immediately and remaining elevated for up to two minutes. They explain that these effects occur because vibration stimulates neuromuscular reflex activity, allowing for force to be generated more rapidly during jumping, while peak force, sprint speed, and change in direction performance remain unaffected. Importantly, these benefits were observed in elite male players, indicating that training level influences responsiveness to such warm-up types.

In contrast, static stretching alone showed limited acute performance benefits. In collegiate female players, static stretching improved agility but did not enhance vertical or horizontal jump performance, whereas dynamic warm-up produced clear gains in jumping ability [21]. Similarly, Barbosa et al. [27] reported that a single session of static stretching, whether performed alone or combined with warm-up, did not affect jump height, power, sprinting, or agility in college volleyball athletes. In youth players, stretching was the only warm-up condition that failed to improve CMJ performance compared with no warm-up [16]. Together, these findings support biomechanical models suggesting that static stretching does not acutely enhance explosive performance, likely because it reduces the ability to produce force quickly during explosive movements.

Foam rolling and vibration foam rolling produced largely negligible performance effects. Popelka, et al. [23] reported no significant differences between dynamic stretching, foam rolling, or their combination in youth players, while Tsai and Chen [19] found that standard foam rolling improved drop jump performance compared with vibration foam rolling or passive rest. However, this latter finding appears isolated, and the broader pattern suggests that foam rolling primarily affects range of motion and perceptual outcomes rather than neuromuscular activation. As such, foam rolling alone does not appear sufficient to enhance explosive volleyball performance.

Several studies reported no significant differences between warm-up types, particularly in younger athletes [16,23,27]. These null findings should be interpreted cautiously, as sample sizes were small (*n* = 8–11), limiting statistical power and increasing the likelihood of Type II error. In some cases, warm-up still outperformed no warm-up [16], whereas in others, no differences were found across conditions [23,27]. This suggests that part of the inconsistency in the literature may reflect methodological limitations rather than a true absence of physiological effects.

Importantly, warm-up was consistently superior to no warm-up across nearly all included studies. Bungkong et al. [17] demonstrated that any warm-up improved jumping ability compared with no warm-up, while Cieśluk et al. [22] showed improved reaction time following a RAMP warm-up compared with baseline. These findings underline that entering explosive activity without proper preparation compromises neuromuscular readiness and mechanical efficiency, increasing both performance decrements and potential injury risk [1,2].

Methodological heterogeneity remains a key limitation. Warm-up duration, intensity, exercise selection, and outcome measures varied substantially across selected studies, complicating direct comparisons. While most studies showed acceptable overall risk of bias, the frequent absence of preregistration and small sample sizes limits confidence in some findings. Nevertheless, consistent patterns emerge when warm-ups are classified according to their physiological intent rather than their specific exercises.

From a practical standpoint, these findings suggest that volleyball warm-ups should prioritize temperature elevation, neuromuscular activation, and movement specificity. Dynamic and resisted components should form the core of preparation, with volleyball-specific movements included to ensure transfer to performance. Static stretching and foam rolling may be used selectively, but they should not replace activation-based warm-ups, and sequencing appears important, with stretching performed after, rather than before, general warm-up activities.

Several limitations of the present review should be acknowledged. The literature search was conducted across PubMed and Google Scholar, which together provide broad coverage of sport science and biomedical literature; however, additional databases such as Scopus, Web of Science, and SPORTDiscus were not included, and some relevant studies may therefore have been missed. The search terms were kept focused on warm-up and volleyball performance, which reflects the scope of the review, though future searches could explore broader terminology to capture additional evidence. As this was a narrative review, prospective registration was not required, though it is noted as a recommended practice for systematic reviews. The included studies varied considerably in warm-up protocols, participant characteristics, and outcome measures, which reflects the diverse approaches used in the field but also makes direct comparisons difficult. Finally, with 13 studies meeting the inclusion criteria, the evidence base remains limited, and findings should be interpreted with appropriate caution until further research is available.

## 5. Conclusions

This systematic review suggests that warm-up strategies may meaningfully influence volleyball performance, particularly for explosive actions such as jumping and agility. Warm-ups that emphasize dynamic movement, neuromuscular activation, resistance, or volleyball-specific actions generally produce greater performance benefits than static stretching, foam rolling, or passive preparation. Static stretching and foam rolling alone provide limited acute performance benefits and should not be relied upon as primary warm-up strategies for volleyball.

Performance improvements tend to be more evident in trained and elite athletes when warm-ups include higher intensity, whereas youth athletes show more variable responses. Overall, warm-up is superior to no warm-up in most performance contexts, reinforcing its role as a necessary component of volleyball preparation rather than an optional one.

Despite these findings, volleyball still lacks a standardized, evidence-based warm-up model comparable to those established in other team sports. Future research should prioritize randomized designs, larger samples, clear intensity prescriptions, and volleyball-specific performance outcomes, particularly for upper body actions such as spiking and serving. Establishing a validated, sport-specific warm-up remains a key objective for optimizing volleyball performance.

## Figures and Tables

**Table 1:** PICOS eligibility criteria used for study selection.

Parameter	Inclusion	Exclusion
Population	Volleyball players (any age, sex, or competitive level)	Non-volleyball athletes
Intervention	Any warm-up protocol (dynamic, static, ballistic, plyometric, PAPE, foam rolling, WBV, sport-specific, etc.) clearly described as a pre-performance intervention	Warm-up used as part of a broader training program rather than as a pre-performance protocol
Comparator	Alternative warm-up type	—
Outcomes	Volleyball-relevant physical or technical performance: vertical jump height, CMJ, approach jump, agility/change in direction, sprint performance, spike or serve speed	Studies focused exclusively on injury prevention without measuring performance outcomes
Study Design	Peer-reviewed research, with full text available	Review papers, meta-analyses, conference abstracts, case reports

**Table 2:** Characteristics of included studies.

Nr.	Study	n	Level of Players	Warm-Up Protocol	Results
1	Tsai and Chen [19]	16	Collegiate male, high-level university athletes	Foam Rolling Exercise (FRE) vs. Vibration Foam Rolling Exercise (VFRE) vs. Control (static rest)	Foam rolling was superior to vibration foam rolling and control for drop jump performance
2	Çilli, et al. [15]	20	Competitive male	Resisted dynamic warm-up (loaded jumps/dynamic activity) vs. Standard/non-resisted condition	Resisted dynamic warm-up significantly increased jump performance
3	Barbosa, et al. [27]	11	College athletes	Control vs. Active warm-up vs. Static stretching vs. Warm-up then Stretching vs. Stretching then Warm-up	No significant differences in EMG activity, jump height, power, agility, or speed
4	Wu, et al. [26]	20	Elite male	Whole body vibration (1-min on platform: 30 Hz, 2 mm displacement) vs. Baseline (pre-warm-up)	WBV warm-up was best for agility and speed–strength
5	Cieśluk, et al. [22]	18	Youth players	RAMP warm-up vs. No warm-up	Reaction time significantly improved after RAMP warm-up compared to before
6	Boughattas, et al. [21]	48	Amateur players (2–3 years competitive experience)	Dynamic warm-up vs. Static stretching vs. No warm-up (control)	Dynamic warm-up: +3.5% vertical jump, +4.2% horizontal jump; Static stretching: improved agility only
7	Bungkong, et al. [17]	16	University-level	No warm-up vs. Jumping rope vs. Conventional jump warm-up	Both warm-up conditions significantly outperformed no warm-up; no significant difference between warm-up types
8	Taş, et al. [24]	24	Competitive female	Routine warm-up vs. Routine + lower-extremity joint distraction exercises (elastic bands)	Combined protocol produced significant improvements in flexibility, ankle dorsiflexion ROM, vertical and horizontal jump performance
9	Alipasali, et al. [20]	27	Recreational male	Dynamic stretching (3×/week, 6 weeks) vs. Static stretching (same frequency) vs. Control (no stretching)	Both static and dynamic stretching were superior to control; no clear superiority between the two stretching types
10	Ünver, et al. [25]	30	14 elite + 16 sub-elite female	Short-term high-intensity warm-up vs. Traditional warm-up vs. No warm-up	Short-term high-intensity warm-up produced significantly greater vertical jump height
11	Popelka, et al. [23]	8	Youth players	Dynamic Stretching vs. Foam Rolling vs. Combination	No significant differences between dynamic stretching, foam rolling, and combined protocol on any performance measure
12	Saez Saez de Villarreal, et al. [18]	12	Competitive adults	WP1: 3 × 5 loaded jumps; WP2: 2 × 4 @ 80% + 2 × 2 @ 85% 1RM; WP3: 2 × 4 @ 80% + 2 × 2 @ 90% + 2 × 1 @ 95% 1RM; WP4: 3 × 5 drop jumps; WP5: Specific volleyball match warm-up; WP6: 3 × 5 @ 30% 1RM; Control: No active warm-up	Best drop jump height: WP3 (≈5.47%), WP5 (≈4.49%), WP1 (≈4.18%), WP2 (≈2.98%); Best CMJ power: WP2 (11.39%), WP5 (10.90%), WP3 (9.00%), WP1 (2.47%)
13	Rezende, et al. [16]	8	Youth competitive	Static stretching vs. Cyclo-ergometer vs. Resistance exercise (leg press) vs. Specific vertical jumping warm-up vs. Control	Specific vertical jumping warm-up produced the highest CMJ; all warm-ups except static stretching were significantly better than control

Note: CMJ = countermovement jump; WBV = whole-body vibration; RAMP = Raise, Activate, Mobilize, Potentiate; FRE = foam rolling exercise; VFRE = vibration foam rolling exercise; EMG = electromyography; 1RM = one-repetition maximum; ROM = range of motion; WP = warm-up protocol.

## Data Availability

All data analyzed are contained within this article.

## Supplementary Materials

The following supporting information can be downloaded at: https://www.mdpi.com/article/10.3390/sports14060218/s1, File S1: PRISMA 2020 Checklist [35].
